# Adequate Knowledge of Stroke Symptoms, Risk Factors, and Necessary Actions in the General Population of Southern Poland

**DOI:** 10.3390/brainsci10121009

**Published:** 2020-12-18

**Authors:** Ewa Krzystanek, Agnieszka Krzak-Kubica, Maciej Świat, Weronika Galus, Justyna Gawryluk

**Affiliations:** 1 Department of Neurology, Faculty of Medical Sciences in Katowice, Medical University of Silesia, Katowice, ul. Medykow 14, 40-752 Katowice, Poland; weroni7@gmail.com (W.G.); jgawryluk@sum.edu.pl (J.G.); 2 Department of Neurology, University Central Hospital, Katowice, ul. Medykow 14, 40-752 Katowice, Poland; agukrzak@gmail.com; 3 Jan Dlugosz University, ul. Waszyngtona 4/8, 42-200 Czestochowa, Poland; maciejswiat@gmail.com

**Keywords:** stroke, stroke risk factors, stroke symptoms, stroke awareness, stroke knowledge, health education

## Abstract

Background and objectives: Stroke is the third most common cause of mortality in developed countries and the primary cause of neurological disability in adults. Recombinant tissue plasminogen activator administered intravenously within 4.5 h from the onset of symptoms constitutes a gold standard in the treatment of acute ischemic stroke. Prompt hospital admission is the prerequisite of effective thrombolysis. Therefore, stroke awareness in the general population is the key factor in timely recognition of the acute stroke victims and determines proper actions. Therefore, the purpose of this study was to determine the awareness of stroke in the general population of the Silesian voivodeship, the most populated region of Poland. We assessed also the “adequate knowledge of stroke”, a combined measure of the optimal level of stroke awareness, as a prerequisite for effective stroke management, and aimed to identify most impacting factor for adequate stoke knowledge, to help shaping education strategies. Materials and Methods: A proprietary anonymous questionnaire consisting of 15 items related to stroke was used in this research. A total of 1134 individuals were surveyed. Additionally to the knowledge of individual aspects of stroke, we assessed “adequate knowledge of stroke”, which was combined measure of risk factors, symptoms, and actions in the case of acute stroke. Results: The accurate definition of stroke was selected by 834 participants (73.5%). The vast majority of them indicated that a stroke is an emergency (92.8%) and medical assistance is required (97.5%). However, 42.4% of respondents did not know any specific symptom of stroke and only 38.6% participants were able to list two or more risk factors, which resulted in only 36.3% of individuals with adequate knowledge of stroke. Education duration, previous occurrence of stroke in relatives or friends, gender and place of residence were identified as independent predictors of adequate knowledge of stroke. Conclusions: 1. Knowledge of stroke in the population of southern Poland is low and may be considered insufficient to address the needs of timely management in the action chain. 2. Previous occurrence of stroke in the relatives or friends is the most impacting factor for adequate knowledge of stroke.

## 1. Introduction

Stroke is the third most common cause of mortality in developed countries and the primary cause of neurological disability in adults [1,2]. Due to the ageing of the worldwide population, it is estimated that in the near future, stroke will be the primary cause of lost years of life [3]. Every year, stroke affects 60,000 people in Poland [4], of which up to 40% die within the first month [5], and one-third die within the first year afterwards [2]. 

Efficacy of acute stroke treatment is time-dependent. Recombinant tissue plasminogen activator (t-PA) for acute ischemic stroke is currently a gold standard in stroke management, but requires administration within 4.5 h from the onset of symptoms [6]. Mechanical thrombectomy, indicated in the acute treatment of ischemic strokes caused by an emergent large-vessel occlusion (ELVO), may be performed within 6 h in case of anterior circulation infarcts. Studies show, that interventions are more beneficial, if performed early. Delayed arrival to the hospital is, therefore, the primary obstacle for the effective treatment [7,8,9]. The main reason for this delay is the lack of public awareness of stroke, its symptoms, risk factors or appropriate behaviors (e.g., immediate emergency call). This problem has been identified in many countries, but in Poland, only two studies were published [10,11,12,13,14,15,16,17,18]. Better understanding of perceived risk factors and warning signs of stroke would facilitate health interventions aimed at reducing stroke-associated morbidity and mortality.

Therefore, the purpose of this study was to determine the awareness of stroke in the general population of the Silesian voivodeship, the most populated region of Poland. We assessed also the “adequate knowledge of stroke”, a combined measure of the optimal level of stroke awareness, as a prerequisite for effective stroke management, and aimed to identify most impacting factor for adequate stoke knowledge, to help shaping education strategies.

## 2. Materials and Methods

This was a cross-sectional sampling investigation study of a survey of adult inhabitants from the Polish region of Silesia, with the population around 4.5 million. This survey was carried out by medical students from the Students’ Scientific Organization of the Department of Neurology (at the Medical University of Silesia, Katowice, Poland) and the authors over a period of 2 years (2011–2012). The sample structure, to be most representative for the population of interest, was defined based on demographic analysis published in 2010 by Statistical Office in Katowice, Poland [19]. The participants were recruited from several places: private homes, waiting rooms of various clinics, cultural centers, and various artistic and cultural events as well as Third Age Universities. The subject and objectives of the study were clarified prior to the interview to each of participants, and oral consent was obtained. Additionally to the general population studied sample, we questioned medical students from the first and sixth year of the medical school, but the collected data are not presented here, as the purpose and objectives were different and may bias the results.

We study used an anonymous, original, 15-item questionnaire to ascertain participant knowledge of stroke. The questionnaire was developed based on FAST test criteria (F-face, A-arm, S-speech, T-time) and available sociological tests used in population-based studies of stroke. The questionnaire consisted of two sections gathering personal data and stroke knowledge.

In the first part, sociodemographic data such as age, gender, level of education, profession, and place of residence (urban vs. rural), as well as previous occurrence of stroke in the family or close friends were collected. For the purpose of the analysis, participants were categorized by age (<45 years and ≥45 years of age) and by level of education (<10 years and ≥10 years of education).

The second part consisted of three open-ended and twelve closed-ended questions pertaining to the definition of stroke, risk factors, symptoms of stroke, action taken in case of stroke, or healthy behaviors to reduce the risk of stroke, as well as information sources, which an individual would use to inform himself or herself. Open-ended questions are considered to better represent actual knowledge of respondents; therefore, in the questionnaire they were asked before the close-ended questions, to avoid prompting the answers. A copy of the questionnaire may be requested from the corresponding author.

For the purpose of the analysis, all answers to the open-ended questions were grouped into specific categories, in order to represent responses typical for the respective question or topic. In case of ambiguity, trained neurologist with more than 10 years of experience in stroke management categorized the responses to best match the defined criteria. Answers directing to several different categories, or clearly out of the scope of questionnaire were grouped in the “other” category. The response to the question about the definition of stroke was considered correct, if in the close-ended question of multiple choice the participant selected either “a condition when a blood vessel within the head bursts and bleeds into and around the brain”, or “a condition when a blood clot blocks the flow of blood in the artery in the brain”, or both. If any other answer was selected, the whole response was considered incorrect. Responses “I don’t know” or missing, e.g., if the participant did not provide any answer to single question or left the space blank, were combined together as incorrect.

Adequate knowledge of stroke was defined based on previously proposed criteria by Ramirez-Moreno et al. [20,21], with minor modifications adopted by consensus. The participants were considered as presenting “adequate knowledge of stroke”, if they correctly named all three:

- at least 1 of 5 major symptoms: consciousness disturbances, speech disturbances, face or mouth asymmetry, paresis or paralysis, or sensory symptoms,
- at least 1 of 11 main risk factors: high blood pressure, smoking, physical inactivity, obesity, alcohol abuse, poor diet/nutrition, hypercholesterolemia, diabetes, cardiovascular diseases, old age, genetic/family history
- call to emergency as a proper action in the case of the acute stroke.

Statistical analysis was performed with use of SYSTAT 12 software (SPSS science, Chicago, IL, USA). Summary data were presented as numbers or proportions of studied group or feature. To analyze differences between the studied groups, chi-square test or the Fisher exact test for categorical data and t-test or Mann–Whitney U test for continuous data were used. For ordinal variables and those with more than 2 categories, we used the Cochran–Mantel–Haenszel test to analyze trends. A univariate analysis was performed to compare ‘‘adequate knowledge of stroke” with demographic and socioeconomic information, as well as previous experience with the disease. Binary logistic regression model was constructed to identify independent factors that may explain the ‘‘adequate knowledge of stroke’’ (dependent variable). Variables were selected using stepwise backward elimination, using *p* greater than 0.15 as exclusion and *p* less than 0.05 as inclusion criteria. A *p* value below 0.05 was considered significant.

## 3. Results

A total of 1134 questionnaires were collected, of which 752 were completed by women (66.3% of all respondents). The mean age (±SD) of the participants was 42.6 ± 15.3 years (ranging between 23–79 years) and the mean age of men was higher than women (*p* = 0.001). City inhabitants under the age of 45 years, with at least 10 years of school education constituted the majority of respondents and proportion of women with higher education level was greater than men (*p* < 0.001). Among the professional groups, the most represented were white-collar workers and retired professionals/pensioners. The most common sources of stroke-related information were television and the press. Less than one-fifth of the participants received stroke-related information from a doctor and 6.4% of the participants have never come across any stroke-related information. Detailed demographic characteristics and the source of stroke-related information are presented in the Table 1. 

Correct definition of stroke was selected as an answer to a close-ended question by 834 (73.5%) participants and place of residence, education duration, or previous stroke in relatives or friends determined differences between subgroups (Table 2). The vast majority of participants indicated that a stroke is an emergency, which requires hospitalization (92.8% and 97.5%, respectively). Stroke was more often considered an emergency by women than men (*p* = 0.003) and individuals with higher education level (*p* = 0.01), but the need for hospitalization was identified equally often in all groups. A call to emergency services, as a response to a close-ended question, considered the only correct action in case of acute stroke, was selected by 82.6% participants, where those with higher education level and previous experience of stroke in relatives or friends were more likely to call an ambulance (*p* = 0.017 and *p* < 0.001, respectively) (Table 2).

Suspected risk factors provided by participants to an open-ended question were sorted in 12 main categories and “other” category for unclassifiable responses (Table 3). 860 (76.8%) participants were able to name at least one risk factor, while 438 (38.6%) listed two or more risk factors. The most frequently mentioned risk factor was old age (55.3%), which was almost twice as frequent as high blood pressure (29.2%) and almost five times more frequent than stress (13.1%). Hypercholesterolemia, diabetes, and genetic/family history were listed by less than 3% of all the respondents. Gender, place of residence and education level and previous stroke in relatives or friends were associated with differences in proportions of identified risk factors between groups. Women, better educated individuals, those living in the city and who had previous contact with stroke listed more risk factors than their counterparts. 274 (24.2%) participants did not provide any response, or selected “I don’t know”.

Suspected symptoms of stroke provided by participants to an open-ended question were sorted in eight main categories and “other” category for unclassifiable responses (Table 4). 653 (57.6%) participants were able to name at least one sign or symptom, while 399 (35.2%) listed two or more risk factors. The most frequently listed symptoms included the consciousness disturbances (33.4%), headaches (26.2%) and paresis (19.5%). The least frequently named symptom was face/mouth asymmetry (3.3%) (part of FAST test), whereas vision disturbances or vision loss were not mentioned at all, thus not included in the table. Similarly to risk factors, gender, place of residence, education duration and previous stroke in relatives or friends were associated with differences in proportions of identified symptoms between subgroups and also here, women, better educated individuals, those living in the city and who had previous contact with stroke listed more symptoms than their counterparts. 481 (42.4%) participants did not provide any response, or selected “I don’t know”.

Stroke prevention strategies were listed in a closed-ended question. The most often selected answers were healthy lifestyle, blood pressure monitoring and smoking cessation (77.3%, 68%, and 60%, respectively). Almost 60% of respondents indicated that regular physical activity is beneficial too, whilst 23.4% of respondents indicated that use of aspirin may reduce the risk of stroke. For this item, gender was not associated with any differences in responses, however, older, better educated individuals and those living in the city listed more stroke symptoms than their counterparts (Table 5).

Adequate knowledge of stroke, as defined in the Methods section above, was assessed as a combination of three aspects: knowledge of major risk factors, recognition of most typical symptoms and a call to emergency services as a proper action. Risk factors and actions were recognized well by all participants (74.1% and 82.6% participants, respectively), but knowledge of stroke symptoms was substantially lower (47.4%), what was probably translated to low overall proportion of participants with adequate knowledge (36.2%) (Table 6). Place of residence, education level, previous stroke in relatives or friends and to some degree gender, contributed to differences between subgroups in proportions of participants correctly recognizing stoke. Women, better educated individuals, those living in the city and who had previous contact with stroke know all critical aspects of stroke better than their counterparts.

In the univariate analysis, female gender (OR = 0.606, 95% CI (0.465, 0.790), *p* = 0.001), living in the urban area (OR = 1.757, 95% CI (1.350, 2.287), *p* < 0.001), better education level (OR = 2.056, 95% CI (1.517, 2.787), *p* < 0.001), and previous occurrence of stroke in relatives or friends (OR = 2.998, 95% CI (2.298, 3.910), *p* < 0.001) were statistically significant predictors of the adequate knowledge of stroke. However, age was not statistically significant predictor of the adequate knowledge of stroke, either considered as a continuous variable, or as a binary age groups (*p* = 0.073 and *p* = 0.134, respectively). In the multivariate stepwise binary logistic regression, education duration, and previous occurrence of stroke in family or friends were identified as strongest independent predictors of adequate knowledge of stroke (both *p* < 0.001) (Table 7). Gender and place of residence were also statistically significant predictors in the model, but age (in years) showed only a trend toward significance *(p* = 0.053).

## 4. Discussion

Identifying the main risk factors for cerebrovascular disease and the knowledge of its major symptoms directly translates into stroke prevention and medical intervention, including thrombolysis. Our research regarding public awareness of stroke is the first in Poland carried out in such a large population. The work is based on a survey, mainly without the suggestion of an answer, which is a big value regarding already well-established knowledge and the way in which respondents reply. The advantage of the survey was the form of open questions, also used in several other publications [5,8]. 

In our study, 834 respondents chose a correct stroke definition (73.5%). In the study by Neau et al. [22], the majority of respondents (76.9%) indicated the brain as an organ affected by stroke. Similar results (85%) were obtained by Mikulik et al. [23] but a knowledge of definition of stroke is not the same as awareness of stroke symptoms and risk factor, so it is definitely not enough to prevent stroke or give an appropriate reaction to a hypothetical case of stroke. The vast majority of our respondents indicated that stroke is an emergency and requires hospitalization (92.8% and 97.5%, respectively). There were 82.6% of respondents who would call an emergency if stroke was suspected, while 13.8% would see their GP, and only 6% would wait for the symptoms to resolve. As shown by Joes et al. [3], published data is divergent in various publications, with 27% up to 100% of respondents declaring that they would call an ambulance in the first place if stroke is suspected, 3% to 40% who would see their GP, and 1% to 10% who would not take any action. 

In our study 57.6% of the participants were able to list at least one symptom, while 35.2% respondents listed two or more symptoms. Respondents were asked to list all known symptoms of stroke. The women, better educated individuals, those living in the city and who had previous contact with stroke listed more symptoms than their counterparts. The ability to name one symptom of stroke differed significantly between studies ranging between 25% and 100% [3]. Just as in the case of the risk factors, stroke symptoms were less recognized if open-ended questions were used. In one study, only 38% of participants were able to identify one or more symptoms when open-ended questions were asked, compared to 100% when a list of possible symptoms was shown [24]. In the largest European study (11.827 Spanish adults), Lundelin et al. (2012) reported that over 11% individuals could not list any symptom of stroke and one in five people indicated that they would do something other than call an ambulance if they think that someone has a stroke [6]. In our study 42.4% of the respondents haven’t known any stroke symptoms and the most frequently listed are: consciousness disturbances (33.4%) and headache (26.2%). Muscle weakness was reported by 19.5% and speech disturbances by 10.6% of the individuals. In other studies, the respondents most often mentioned paresis, speech disorders and dizziness [22,25,26,27]. On the other hand, vision impairment, diplopia, and gait disturbance were mentioned rare (<5%), in the presented study not once. Masztalewicz at al. studied the percentage of callers (potentially every respondent in our study) who recognized stroke symptoms in the Polish West Pomerania. In similar period of time (2013–2014) correct answer listed 39.9% individuals, who called emergency because noted stroke symptoms [18]. 

In the publication Teuschl et al. (2010) regarding the impact of the awareness of stroke factors on delayed transport to the hospital highlighted the weak impact of education and sociodemographic data, such as age or residential area on the reaction taken after the onset of symptoms of stroke, and only 25–56% of patients recognized symptoms of stroke [4]. The disadvantages of the survey was to list disturbances of consciousness and headaches among symptoms associated with stroke, because the symptoms are not typical of acute ischemic stroke. 

Awareness of stroke symptoms was better but not perfect among Emergency Medical Services (EMS) employees in Dubai: 79% of EMS staff were able to name the symptoms of a stroke [5].

In our study 24.2% of the respondents did not name any stroke risk factor, 39% were able to name at least one of them, 20% named two risk factors, and 10% were able to name three or more. In the study by Gongor-Rivera et al. [28] it was 33.3% (none), 29.4% (at least 1 factor), 25.2% (two or more factors), and only 12.1% (three or more factors), respectively. The proportion of individuals naming one or more stroke risk factors differed significantly between studies ranging between 18% and 94% for open-ended questions and between 42% and 97% for closed-ended questions [3]. In a large urban population in France, the majority of patients (62.3%) were able to name three risk factors for stroke, but omitted the most important of them, and less than half of the respondents were able to recognize one or more warning signs. They based their knowledge on magazines and books, as well as on the experience of families in whom stroke occurred [22]. In our study not press but television was the main source of information of stroke. 

The stroke risk factors mentioned most often by our respondents were an old age (55.3%, but it was separate question in opposite to the cited below), then hypertension (29.2%), smoking (10.8%), cardiovascular disease (8.6%), and stress (13.1%). Lipid disorders, diabetes, obesity, and genetic factors were mentioned by less than 5% of all respondents. In other studies, hypertension and smoking were the most frequently mentioned risk factors for stroke, whereas diabetes, obesity, heart disease (including cardiomyopathy and atrial fibrillation), using oral contraceptives, and old age were mentioned by less than 5% of individuals [1,22,23,24]. In our study, in the closed-ended question on stroke prevention strategies, a healthy lifestyle and regular blood pressure control were chosen as the best prevention (77% and 68%, respectively). 60% of our respondents also indicated smoking cessation and 58% regular physical activity. 

In Table 8 some data regarding stroke awareness received from different population were compared. The methods used in these study, cultural conditions and interpretation of results are different but we can say that in Poland the awareness of stroke is similar as in the rest of the world, but not sufficient. The only significant difference seems to be the symptom of stroke most frequently reported by the respondents. However, such differences are not visible in the case of the most frequently mentioned risk factors of stroke. 

Our arbitrary definition of “adequate stroke knowledge” is based on Ramirez-Moreno et al. and Segura et al., but a little bit more conservative (just five main typical stroke symptoms) [20,21]. In the first mentioned paper, 40% of the respondents have adequate stroke knowledge, which is comparable to our study (36.2%). We also confirmed, that poorly educated people have less knowledge, but not older people. So the situation is quite similar in Poland and in Spain [20]. “I call an emergency” was answered by 82.6% in our group, but 24.2% of the people did not list one risk factor, and 42.4% did not know one stroke symptom. According the authors of this study such a general stroke awareness is not sufficient to improve a rate of thrombolysis or to favor the healthy behaviors. Because the most respondents (in our study 58.7%, in Ramirez-Moreno at al. study 53.6%) named TV as an source of stroke information, it means that TV campaign or advertisements are not focused on proper issues and should be more informative. According to our results” stroke in relatives or friends’ is the strongest independent predictors for adequate stroke knowledge, therefore media should be encouraged to provide sufficient and more detailed information focused on virtual stroke presentation. This may help to shape education campaigns referring to close relatives, or even provide virtual patient for the purpose of personification of stroke victim to be more impactful and emotional. It is known, that the information processed during emotional situations is remembered for very long time.

Early recognition of stroke symptoms is the key to maximizing the opportunity for medical intervention, in particular thrombolysis. Our study highlights some gaps in common knowledge of strokes. We need to improve public awareness of stroke in Poland. In our study only 13% of the respondents heard something about stroke whereas social campaign on cervical cancer prevention reached 79% of them. Currently, campaigns are in progress in Poland under the auspices of the Polish Neurological Society (stop strokes, prevent.pl, time is important, and open hand after stroke), the aim of which is to spread awareness of stroke prevention and diagnosis and post-stroke assistance. Public campaigns, as shown in research [30,31,35], are among the best ways to achieve this. In the Polish papers, Kobayashi et al. (2018) and Masztalewicz et al. (2016) emphasizes that despite very low quality of evidence, educational campaigns to increase the awareness of immediately calling emergency medical services are strongly recommended [7,18]. 

The presented study has some limitations. First, the disadvantage of the work was the construction of the questionnaire which ignored time as a risk factor for the consolidation of neurological symptoms. Current ongoing campaigns are focused on the time factor in the development of stroke symptoms and a key factor in the management of acute stroke, in particular thrombolysis. Second, using the "don’t know" option in open-ended questions may have caused more respondents to choose it without thinking about the right answer. Thus, a large number of undecided subjects may not be indicative of a failure to recognize stroke symptoms, but may be caused by the lack of willingness to fill in the questionnaire reliably. Perhaps, therefore, the "don’t know" option should have been removed from the questionnaire, which would have prompted a greater number of respondents to provide their own answers to open-ended questions.

## 5. Conclusions

1. Knowledge of stroke in the population of southern Poland is low and may be considered insufficient to address the needs of timely management in the action chain. 2. Previous occurrence of stroke in the relatives or friends is the most impacting factor for adequate knowledge of stroke.

## Figures and Tables

**Table 1:** Group characteristics and source of information about stroke.

All		Total (*n*%)		Men (*n*%)		Women (*n*%)		*p*
		1134	100%	382	33.7%	752	66.3%	
Age, years	mean (±SD)	42.6	(15.3)	44.7	(15.6)	41.5	(15.1)	0.001
	median (min–max)	37	(23–79)	39	(24–75)	36	(23–79)	
Age group	<45 years	703	62.0%	215	30.6%	488	69.4%	0.005
	≥45 years	431	38.0%	167	38.7%	264	61.3%	
Place of residence	urban	735	64.8%	239	32.5%	496	67.5%	0.258
	rural	399	35.2%	143	35.8%	256	64.2%	
Education duration	<10 years	281	24.8%	131	46.6%	150	53.4%	<0.001
	≥10 years	853	75.2%	251	29.4%	602	70.6%	
Profession	white-collar worker	452	39.9%	116	25.7%	336	74.3%	<0.001
	retired	273	24.1%	109	39.9%	164	60.1%	
	manual worker	225	19.8%	117	52.0%	108	48.0%	
	unemployed	77	6.8%	15	19.5%	62	80.5%	
	health care worker	74	6.5%	9	12.2%	65	87.8%	
	company owner	33	2.9%	16	48.5%	17	51.5%	
Stroke in relatives or friends		327	28.8%	87	26.6%	240	73.4%	
Source of stroke information								
TV		666	58.7%					
radio		271	23.9%					
press		427	37.7%					
internet		242	21.3%					
doctor’s office		204	18.0%					
other		278	24.5%					
none		73	6.4%					

**Table 2:** Correct stroke definition and proper actions required in case of stroke reported by participants grouped by gender, age group, place of residence, education level, and profession.

	Stroke Knowledge (*n*, %)						Action Taken (*n*, %)					
	Correct Stroke Definition		Stroke Is an Emergency		Requires Hospitalization		Call an Emergency Service		Go to GP		Wait	
All	**834**	**73.5%**	**1052**	**92.8%**	**1106**	97.5%	937	82.6%	157	13.8%	68	6.0%
Gender												
male	287	75.1%	**342**	**89.5%**	368	96.3%	304	79.6%	60	15.7%	29	7.6%
female	547	72.7%	**710**	**94.4%**	738	98.1%	633	84.2%	97	12.9%	39	5.2%
Age group												
<45 years	514	73.1%	658	93.6%	689	98.0%	578	82.2%	97	13.8%	46	6.5%
≥45 years	320	74.2%	394	91.4%	417	96.8%	359	83.3%	60	13.9%	22	5.1%
Place of residence												
urban	**560**	**76.2%**	683	92.9%	720	98.0%	618	84.1%	93	12.7%	43	5.9%
rural	**274**	**68.7%**	369	92.5%	386	96.7%	319	79.9%	64	16.0%	25	6.3%
Education duration												
<10 years	**190**	**67.6%**	**251**	**89.3%**	270	96.1%	**219**	**77.9%**	50	17.8%	23	8.2%
>10 years	**644**	**75.5%**	**801**	**93.9%**	836	98.0%	**718**	**84.2%**	107	12.5%	45	5.3%
Stroke in relatives or friends												
yes	**259**	**79.2%**	308	94.2%	322	98.5%	**293**	**89.6%**	**24**	**7.3%**	15	4.6%
no	**575**	**71.2%**	744	92.2%	784	97.1%	**644**	**79.8%**	**133**	**16.5%**	53	6.6%
Profession												
white-collar worker	346	76.5%	423	93.6%	442	97.8%	391	86.5%	50	11.1%	22	4.9%
retired	183	67.0%	245	89.7%	265	97.1%	223	81.7%	41	15.0%	18	6.6%
manual worker	163	72.4%	206	91.6%	218	96.9%	170	75.6%	42	18.7%	19	8.4%
unemployed	56	72.7%	75	97.4%	75	97.4%	62	80.5%	12	15.6%	4	5.2%
health care worker	61	82.4%	71	95.9%	73	98.6%	62	83.8%	10	13.5%	3	4.1%
company owner	25	75.8%	32	97.0%	33	100.0%	29	87.9%	2	6.1%	2	6.1%

Note: Between-groups comparisons performed with chi-squared test, **bold** indicates *p* < 0.05. Proportions in the Profession category were not analyzed. Responses “Go to GP” and “Wait” were not analyzed separately, as they were the part of multiple choice question pertaining to an emergency action.

**Table 3:** Stroke risk factors provided by participants in response to an open-ended question grouped by gender, age group, place of residence, education level and profession.

	High Blood Pressure	Smoking	Physical Inactivity	Obesity	Alcohol Abuse	Poor Diet/Nutrition	Hypercholesterolemia	Diabetes	Cardiovascular Diseases	Old Age	Stress	Genetic/Family History	Other	Don’t Know/No Response
All														
*n*	331	122	58	34	78	83	25	32	98	627	148	26	38	274
%	29.2	10.8	5.1	3.0	6.9	7.3	2.2	2.8	8.6	55.3	13.1	2.3	3.4	24.2
Gender *														
female, *n*	249	85	37	24	51	62	22	26	71	412	97	16	25	178
%	33.1	11.3	4.9	3.2	6.8	8.2	2.9	3.5	9.4	54.8	12.9	2.1	3.3	23.7
male, *n*	82	37	21	10	27	21	3	6	27	215	51	10	13	96
%	21.5	9.7	5.5	2.6	7.1	5.5	0.8	1.6	7.1	56.3	13.4	2.6	3.4	25.1
Age group														
<45 years, *n*	200	79	37	23	42	50	11	17	69	392	81	9	23	181
%	28.4	11.2	5.3	3.3	6.0	7.1	1.6	2.4	9.8	55.8	11.5	1.3	3.3	25.7
≥45 years, *n*	131	43	21	11	36	33	14	15	29	235	67	17	15	93
%	30.4	10.0	4.9	2.6	8.4	7.7	3.2	3.5	6.7	54.5	15.5	3.9	3.5	21.6
Place of residence *														
urban, *n*	238	92	46	25	60	57	21	25	72	401	109	22	33	159
%	32.4	12.5	6.3	3.4	8.2	7.8	2.9	3.4	9.8	54.6	14.8	3.0	4.5	21.6
rural, *n*	93	30	12	9	18	26	4	7	26	226	39	4	5	115
%	23.3	7.5	3.0	2.3	4.5	6.5	1.0	1.8	6.5	56.6	9.8	1.0	1.3	28.8
Education duration *														
<10 years, *n*	66	20	6	8	13	14	3	2	16	138	30	4	7	86
%	23.5	7.1	2.1	2.8	4.6	5.0	1.1	0.7	5.7	49.1	10.7	1.4	2.5	30.6
>10 years, *n*	265	102	52	26	65	69	22	30	82	489	118	22	31	188
%	31.1	12.0	6.1	3.0	7.6	8.1	2.6	3.5	9.6	57.3	13.8	2.6	3.6	22.0
Stroke in relatives or friends *														
yes, *n*	144	52	23	15	29	45	10	20	44	197	62	11	11	37
%	44.0	15.9	7.0	4.6	8.9	13.8	3.1	6.1	13.5	60.2	19.0	3.4	3.4	11.3
no, *n*	187	70	35	19	49	38	15	12	54	430	86	15	27	237
%	23.2	8.7	4.3	2.4	6.1	4.7	1.9	1.5	6.7	53.3	10.7	1.9	3.3	29.4
Profession														
white-collar worker, *n*	140	52	29	17	32	44	10	20	55	262	59	11	14	101
%	31.0	11.5	6.4	3.8	7.1	9.7	2.2	4.4	12.2	58.0	13.1	2.4	3.1	22.3
retired, *n*	77	21	9	6	19	7	8	5	17	140	40	7	13	73
%	28.2	7.7	3.3	2.2	7.0	2.6	2.9	1.8	6.2	51.3	14.7	2.6	4.8	26.7
manual worker, *n*	48	18	9	5	10	15	0	1	12	118	24	3	4	63
%	21.3	8.0	4.0	2.2	4.4	6.7		0.4	5.3	52.4	10.7	1.3	1.8	28.0
unemployed, *n*	11	8	2	0	4	4	2	1	0	47	6	2	2	24
%	14.3	10.4	2.6		5.2	5.2	2.6	1.3		61.0	7.8	2.6	2.6	31.2
health care professional, *n*	44	17	6	5	9	10	3	5	12	46	11	3	5	6
%	59.5	23.0	8.1	6.8	12.2	13.5	4.1	6.8	16.2	62.2	14.9	4.1	6.8	8.1
private business, *n*	11	6	3	1	4	3	2	0	2	14	8	0	0	7
%	33.3	18.2	9.1	3.0	12.1	9.1	6.1		6.1	42.4	24.2			21.2

Note: Between-groups comparisons performed with Cochran–Mantel–Haenszel test, * indicates *p* < 0.05. Proportions in the Profession category were not analyzed.

**Table 4:** Stroke symptoms provided by participants in response to an open-ended question grouped by gender, age group, place of residence, education level, and profession.

	Consciousness Disturbances	Headache	Speech Disturbances	Face or mouth Asymmetry	Paresis, Paralysis	Sensory Symptoms	Vertigo, Dizziness	Nausea, Vomiting	Other	Don’t Know
All										
*n*	379	297	119	37	221	62	51	67	70	481
%	33.4	26.2	10.5	3.3	19.5	5.5	4.5	5.9	6.2	42.4
Gender *										
female, *n*	269	218	91	25	164	48	33	48	51	289
%	35.8	29.0	12.1	3.3	21.8	6.4	4.4	6.4	6.8	38.4
male, *n*	110	79	28	12	57	14	18	19	19	192
%	28.8	20.7	7.3	3.1	14.9	3.7	4.7	5.0	5.0	50.3
Age group										
<45 years, *n*	218	195	74	16	150	39	35	42	46	297
%	31.0	27.7	10.5	2.3	21.3	5.5	5.0	6.0	6.5	42.2
≥45 years, *n*	161	102	45	21	71	23	16	25	24	184
%	37.4	23.7	10.4	4.9	16.5	5.3	3.7	5.8	5.6	42.7
Place of residence *										
urban, *n*	261	185	88	27	152	50	32	47	56	296
%	35.5	25.2	12.0	3.7	20.7	6.8	4.4	6.4	7.6	40.3
rural, *n*	118	112	31	10	69	12	19	20	14	185
%	29.6	28.1	7.8	2.5	17.3	3.0	4.8	5.0	3.5	46.4
Education duration *										
<10 years, *n*	75	56	13	11	31	12	12	15	6	152
%	26.7	19.9	4.6	3.9	11.0	4.3	4.3	5.3	2.1	54.1
>10 years, *n*	304	241	106	26	190	50	39	52	64	329
%	35.6	28.3	12.4	3.0	22.3	5.9	4.6	6.1	7.5	38.6
Stroke in relatives or friends *										
yes, *n*	142	96	58	21	98	20	16	19	40	80
%	43.4	29.4	17.7	6.4	30.0	6.1	4.9	5.8	12.2	24.5
no, *n*	237	201	61	16	123	42	35	48	30	401
%	29.4	24.9	7.6	2.0	15.2	5.2	4.3	5.9	3.7	49.7
Profession										
white-collar worker, *n*	154	111	61	15	107	29	20	26	32	180
%	34.1	24.6	13.5	3.3	23.7	6.4	4.4	5.8	7.1	39.8
retired, *n*	91	70	24	7	38	16	9	11	9	126
%	33.3	25.6	8.8	2.6	13.9	5.9	3.3	4.0	3.3	46.2
manual worker, *n*	59	53	12	7	28	6	13	16	7	108
%	26.2	23.6	5.3	3.1	12.4	2.7	5.8	7.1	3.1	48.0
unemployed, *n*	24	22	4	4	14	3	1	5	4	40
%	31.2	28.6	5.2	5.2	18.2	3.9	1.3	6.5	5.2	51.9
health care professional, *n*	38	31	15	4	29	7	8	8	14	12
%	51.4	41.9	20.3	5.4	39.2	9.5	10.8	10.8	18.9	16.2
private business, *n*	13	10	3	0	5	1	0	1	4	15
%	39.4	30.3	9.1		15.2	3.0		3.0	12.1	45.5

Note: Between-groups comparisons performed with Cochran–Mantel–Haenszel test, * indicates *p* < 0.05. Proportions in the Profession category were not analyzed.

**Table 5:** Stroke prevention strategies indicated by participants in the response to close-ended question by gender, age group, place of residence, education level, and profession.

	Smoking Cessation	Moderate Alcohol Consumption	Low Fat Diet	Taking Aspirin	Regular Physical Activity	Healthy Lifestyle	Blood Pressure Monitoring	Taking Vitamins
All								
*n*	680	367	559	265	656	877	771	155
%	60.0	32.4	49.3	23.4	57.8	77.3	68.0	13.7
Gender								
male, *n*	226	124	174	84	215	296	249	65
%	59.2	32.5	45.5	22.0	56.3	77.5	65.2	17.0
female, *n*	454	243	385	181	441	581	522	90
%	60.4	32.3	51.2	24.1	58.6	77.3	69.4	12.0
Age group *								
<45 years, *n*	412	214	336	147	405	539	460	88
%	58.6	30.4	47.8	20.9	57.6	76.7	65.4	12.5
≥45 years, *n*	268	153	223	118	251	338	311	67
%	62.2	35.5	51.7	27.4	58.2	78.4	72.2	15.5
Place of residence *								
urban, *n*	456	259	390	193	438	582	525	97
%	62.0	35.2	53.1	26.3	59.6	79.2	71.4	13.2
rural, *n*	224	108	169	72	218	295	246	58
%	56.1	27.1	42.4	18.0	54.6	73.9	61.7	14.5
Education duration *								
<10 years, *n*	148	87	113	50	138	210	182	42
%	52.7	31.0	40.2	17.8	49.1	74.7	64.8	14.9
>10 years, *n*	532	280	446	215	518	667	589	113
%	62.4	32.8	52.3	25.2	60.7	78.2	69.1	13.2
Stroke in relatives or friends *								
yes, *n*	215	121	194	92	219	261	246	37
%	65.7	37.0	59.3	28.1	67.0	79.8	75.2	11.3
no, *n*	465	246	365	173	437	617	525	118
%	57.6	30.5	45.2	21.4	54.2	76.5	65.1	14.6
Profession								
white-collar worker, *n*	277	148	234	99	282	361	300	56
%	61.3	32.7	51.8	21.9	62.4	79.9	66.4	12.4
retired, *n*	157	90	130	76	146	204	193	37
%	57.5	33.0	47.6	27.8	53.5	74.7	70.7	13.6
manual worker, *n*	125	69	88	37	117	166	146	42
%	55.6	30.7	39.1	16.4	52.0	73.8	64.9	18.7
unemployed, *n*	48	20	36	12	37	58	49	10
%	62.3	26.0	46.8	15.6	48.1	75.3	63.6	13.0
health care professional, *n*	55	28	52	28	52	61	59	7
%	74.3	37.8	70.3	37.8	70.3	82.4	79.7	9.5
private business, *n*	18	12	19	13	22	27	24	3
%	54.5	36.4	57.6	39.4	66.7	81.8	72.7	9.1

Note: Between-groups comparisons performed with Cochran Mantel Haenszel test, * indicates *p* < 0.05. Proportions in the Profession category were not analyzed.

**Table 6:** Adequate knowledge of stroke as a combined measure of correct knowledge of risk factors, symptoms and necessary actions by gender, age group, place of residence, education level, and profession.

n, %	Knows at Least One of Risk Factors		Names Correctly at Least One of Typical Signs or Symptoms		Correct Action Taken (Call Emergency)		Adequate Knowledge (Correctly Identified All Three Conditions)	
All	840	74.1%	537	47.4%	937	82.6%	411	36.2%
Gender								
female	564	75.0%	**385**	**51.2%**	633	84.2%	**301**	**40.0%**
male	276	72.3%	**152**	**39.8%**	304	79.6%	**110**	**28.8%**
Age group								
<45 years	510	72.5%	325	46.2%	578	82.2%	243	34.6%
≥45 years	330	76.6%	212	49.2%	359	83.3%	168	39.0%
Place of residence								
urban	**563**	**76.6%**	**376**	**51.2%**	618	84.1%	**299**	**40.7%**
rural	**277**	**69.4%**	**161**	**40.4%**	319	79.9%	**112**	**28.1%**
Education duration								
<10 years	**187**	**66.5%**	**100**	**35.6%**	**219**	**77.9%**	**69**	**24.6%**
≥10 years	**653**	**76.6%**	**437**	**51.2%**	**718**	**84.2%**	**342**	**40.1%**
Stroke in relatives or friends								
yes	**284**	**86.8%**	**213**	**65.1%**	**293**	**89.6%**	**179**	**54.7%**
no	**556**	**68.9%**	**324**	**40.1%**	**644**	**79.8%**	**232**	**28.7%**
Profession								
white-collar worker	345	76.3%	231	51.1%	391	86.5%	186	41.2%
retired	194	71.1%	122	44.7%	223	81.7%	91	33.3%
manual worker	157	69.8%	84	37.3%	170	75.6%	53	23.6%
unemployed	53	68.8%	29	37.7%	62	80.5%	20	26.0%
health care professional	67	90.5%	55	74.3%	62	83.8%	46	62.2%
private business	24	72.7%	16	48.5%	29	87.9%	15	45.5%

Note: Between-groups comparisons performed with chi-squared test, **bold** indicates *p* < 0.05. Proportions in the Profession category were not analyzed.

**Table 7:** Multivariate stepwise binary logistic regression model to identify independent predictors of adequate knowledge of stroke.

Parameter	Values	Estimate	OR (95% CI)	*p*-Value
Gender	Female = 0, male = 1	−0.362	0.696 (0.526, 0.921)	0.011
Age	years	0.009	1.009 (1.000, 1.018)	0.053
Place of residence	Rural = 0, urban = 1	0.358	1.430 (1.077, 1.899)	0.013
Education duration	<10 years = 0, ≥10 years = 1	0.688	1.989 (1.432, 2.763)	<0.001
Stroke in family or friends	No = 0, yes = 1	1.039	2.826 (2.153, 3.710)	<0.001
Constant		−1.914		<0.001

Note: *p*-value of the model <0.001, McFadden’s Rho^2^ = 0.072.

**Table 8:** Some data regarding stroke awareness received from different population.

	% of Subjects Reported at Least 1 Correct Warning Sign of Stroke	The Most Frequent Mentioned Warning Sign of Stroke	% of Subjects Named at Least 1 Correct Risk Factor for Stroke	The Most Frequent Mentioned Risk Factor for Stroke	% of Subjects Calls an Emergency
Krzystanek et al. (Poland)	57.6	consciousness disturbances	76.8	old age, high blood pressure	82.6
Ramirez-Moreno et al. (Spain, 2015) [20]	73.0	paralysis/weakness	59.2	smoking, high blood pressure	80
Hickey et al. (Ireland, 2009) [29]	87.0	slurred speech	94.0	hypertension	no data
\Metias et al. (Canada, 2017) [30]	88.7	weakness, numbness or paralysis	83.1	smoking, high blood pressure	94.1
Nordanstig et al. (Sweden, 2017) [31]	72.0	speech disturbance	no data	smoking, stress	65.0
Akiyama et al. (Japan, 2013) [32]	no data	speech disturbances	no data	high blood pressure, diabetes	67.0
Krishnamurthi et al. (New Zealand, 2020) [33]	98.0	sudden difficulty speaking or understanding others	78.0	high blood pressure	80.0
Jackson et al. (USA, 2020) [34]	no data	face, arm, leg, side numbness	no data	no data	96.3
